# Supplementary material for: Epigenetic Potential and Dispersal Propensity in a Free‐Living Songbird: A Spatial and Temporal Approach
Source: Mol Ecol. 2025 Oct 22;34(22):e70143. doi: 10.1111/mec.70143 (PMC12617058; doi:10.1111/mec.70143)
Supplement: Supplementary file 1 — Figure S1: Mean sequence depth by sex and chromosome type (i.e., autosomes vs. Z chromosome). Figure S2: Epigenetic potential (calculated as the number of CpG + polyCpG polymorphisms) by group category across sampling years. Figure S3: Principal component analysis showing no genetic variant structure linked to dispersal group categories (for details see methods). Figure S4: Principal component analysis showing no genetic variant structure linked to sequencing run (for details see methods). Figure S5: Epigenetic potential (estimated as total number of CpGs across the genome, including variant and non‐variant sites) across the four dispersal group categories included in the study. Table S1: Distribution of CpG and non‐CpG polymorphisms across the genome in the study. [file MEC-34-e70143-s001.pdf]

SUPPLEMENTARY INFORMATION TO:

Epigenetic potential and dispersal propensity in a free-living songbird: a spatial and temporal approach

Blanca Jimeno, Marianthi Tangili, Julio C. Domínguez, David Canal, Carlos Camacho, Jaime Potti, Jesús T. García, Jesús Martínez-Padilla and Mark Ravinet

Table. S1. Distribution of CpG and non-CpG polymorphisms across the genome in the study population.

Variant status	Annotation category	Counts	Proportion
CpG	promoter	38484	0.048044
	exon	68109	0.085027
	intron	328158	0.409673
	intergenic	366273	0.457256
polyCpG	promoter	28485	0.038708
	exon	28806	0.039144
	intron	316108	0.429553
	intergenic	362501	0.492595
non-CpG	promoter	214748	0.035161
	exon	164390	0.026916
	intron	2634894	0.431415
	intergenic	3093534	0.506508

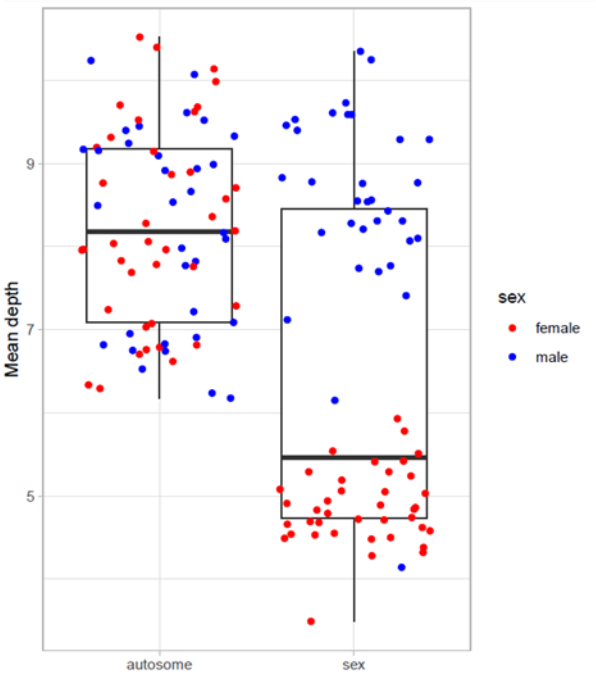

Fig. S1. Mean sequence depth by sex and chromosome type (i.e. autosomes vs. Z chromosome).

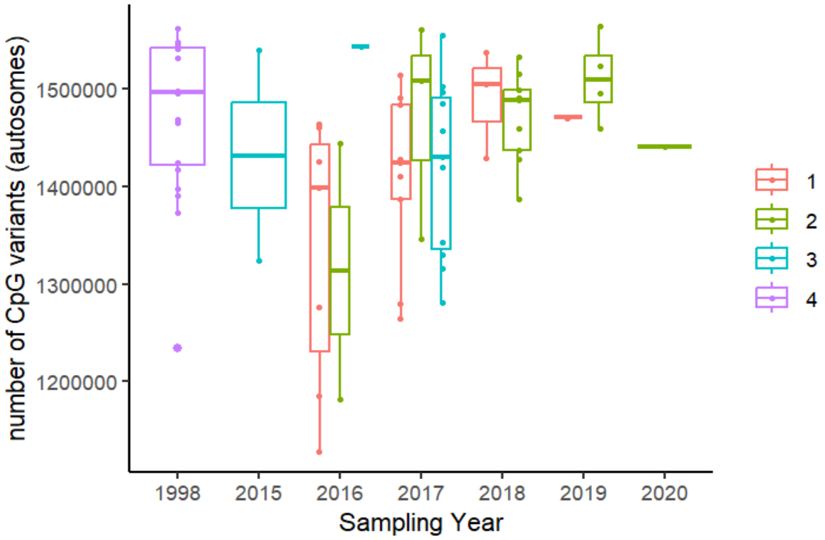

Fig. S2. Epigenetic potential (calculated as the number of CpG + polyCpG polymorphisms) by group category across sampling years. CpG counts in Group 4 sampling year (1998) did not overall differ from CpG counts in those sampling years covered in Group 1. The only significant difference existing among all sampling years was between 1998 and 2016, with lower counts in 2016 (Tukey Test; p=0.025). Year 2016 included samples from group categories 1 and 2 and also tended to have lower CpG count compared to other years (i.e. p < 0.1 for 2016 vs. 2018 and 2016 vs. 2019) due to higher % missingness. This pattern was confirmed by the above differences in sampling years disappearing when including percentage of sequencing missingness as covariate (Tukey Test; all p values > 0.21).

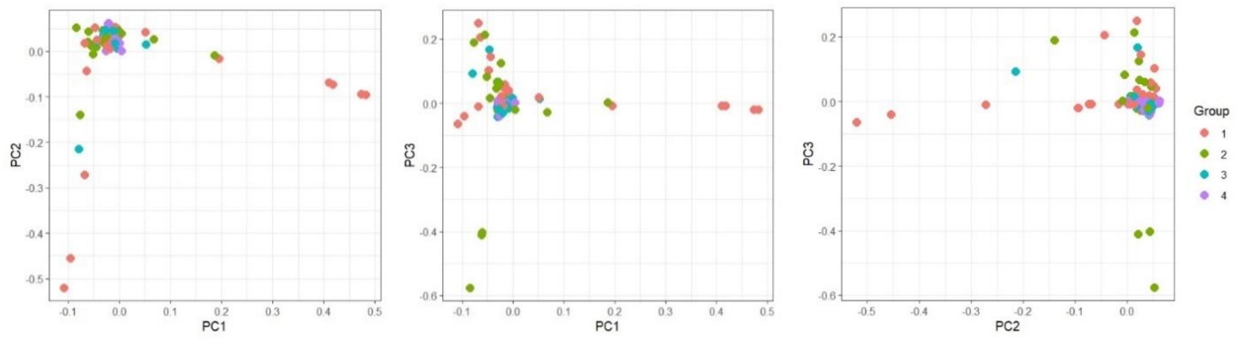

Fig S3: Principal component analysis showing no genetic variant structure linked to dispersal group categories (for details see methods). Although some datapoints (especially from Group 1) appear further from the rest, excluding the individuals most distant in the genetic structure analyses did not affect the results qualitatively.

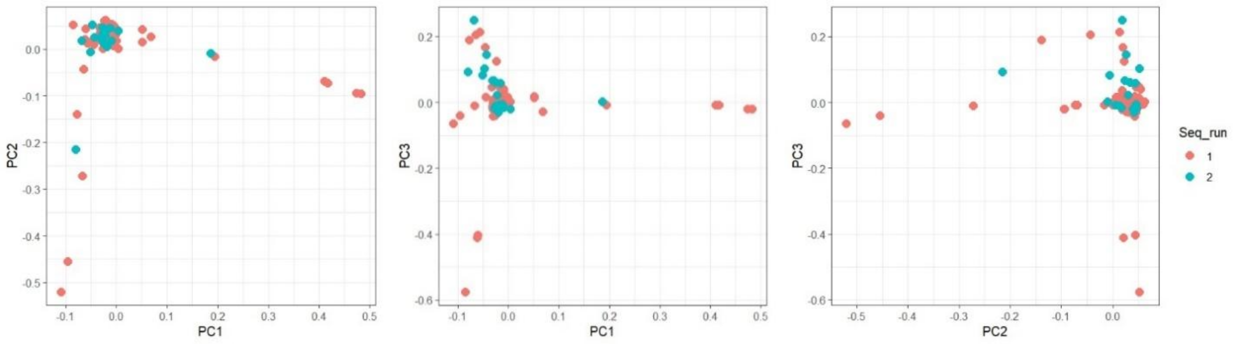

Fig S4: Principal component analysis showing no genetic variant structure linked to sequencing run (for details see methods).

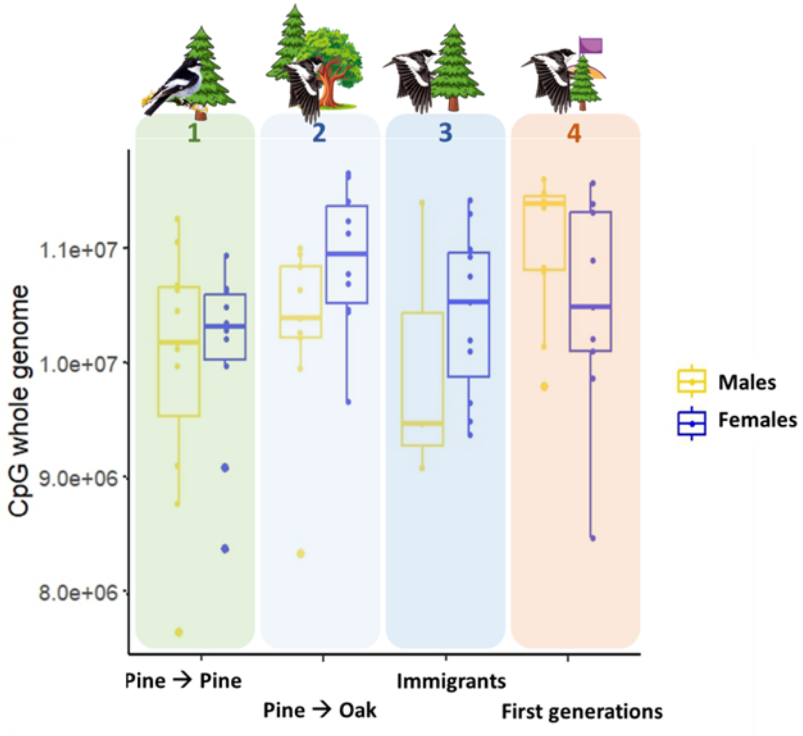

Fig S5: Epigenetic potential (estimated as total number of CpGs across the genome, including variant and non-variant sites) across the four dispersal group categories included in the study. Group categories correspond to: 1. Birds reared and breeding in the pine forest; 2. Birds reared in the pine forest but breeding in the oak forest; 3. Birds identified as immigrants to the population, 4. First generations of birds breeding in the pine forest after patch colonization. The three comparisons tested were made between dispersal categories predicted to have high (blue and orange) and low (green) epigenetic potential, and correspond to different spatial (blue vs. green) and temporal (orange vs. green) scales. The bottom and top lines of the box represent the interquartile range, and the horizontal line inside the box represents the median. The whiskers represent values outside the lower and upper quartile. Note that statistical models included sample missingness as weighing variable (see results).
